# Acquired Ano-Rectal Sinuses and Fistulas Involving Genito-Urinary Structures in Men: A Case Series

**DOI:** 10.7759/cureus.77832

**Published:** 2025-01-22

**Authors:** Abhraneel Das, Devesh Malik, Saurav S Bhadoria, Asmita Asmita

**Affiliations:** 1 Radiodiagnosis, Sanjay Gandhi Postgraduate Institute of Medical Sciences, Lucknow, IND

**Keywords:** ano-rectal sinuses, gastro-radiology, male genito-urinary system, mr fistulography, perianal fistulas

## Abstract

Acquired ano-rectal fistulas and sinuses are frequently observed clinical conditions, with a higher prevalence in men. While these conditions typically remain confined to the ano-rectal region, the potential for involvement of nearby pelvic genito-urinary structures exists due to their anatomical closeness. Interestingly, such cases are rarely documented in medical literature. This could be attributed to the protective role of anatomical barriers, such as pelvic fasciae, which limit the spread of inflammatory or infectious processes from the ano-rectal area to adjacent structures.

In this article, we describe a series of four patients who presented with ano-rectal fistulas and sinuses that extended into pelvic genito-urinary organs. Among them, three were diagnosed with underlying inflammatory bowel disease. The unusual symptoms observed in all cases posed diagnostic challenges, necessitating a thorough evaluation. This series highlights the importance of considering atypical presentations in patients with complex ano-rectal pathologies and underscores the value of multidisciplinary approaches in achieving accurate diagnoses and effective treatment plans.

## Introduction

Acquired ano-rectal fistulas and sinuses are a prevalent condition, with a higher incidence reported in the male population [1]. Surgical management remains the primary treatment approach for most patients. Magnetic resonance imaging (MRI) is an integral part of the preoperative evaluation, offering critical insights into the extent and severity of the disease, thereby facilitating precise surgical planning.

Typically, these cases are identified when patients present with perineal pain or discharging cutaneous openings. However, certain patients may remain asymptomatic or exhibit atypical symptoms, leading to delayed diagnosis and progression to more advanced stages of the disease [2].

Although the anatomical proximity of pelvic genito-urinary organs to the ano-rectal region theoretically predisposes them to involvement, such cases are rarely documented in the literature. This rarity is attributed to natural anatomical barriers, including the mesorectal fascia, fascia of Denonvilliers, Colle’s fascia, Buck’s fascia, and the perineal diaphragm, which act as protective structures preventing the extension of fistulas and sinuses into adjacent pelvic organs.

In this article, we present a series of four cases demonstrating the extension of advanced ano-rectal fistulas and sinuses into pelvic genito-urinary structures. This series highlights the diverse clinical presentations, the role of MRI in diagnosis, and the subsequent surgical management strategies employed in these rare and complex cases.

## Case presentation

Anatomical considerations and etiopathogenesis

The anal canal is surrounded by the internal and external anal sphincters, which consist of smooth and striated muscles, respectively. The external sphincter is posteriorly attached to the ano-coccygeal ligament and anteriorly attached to the perineal body and urogenital diaphragm (and bulbocavernosus muscles in men) and continues proximally with the puborectalis muscle (which marks the ano-rectal junction), which in turn, merges with the levator ani muscles. The internal sphincter is formed by the distal circular muscle of gastro-intestinal tract [3]. The inter-sphincteric space is the plane of surgical dissection between the two sphincters. It consists of loose areolar fatty tissue. The fat-rich ischio-anal fossa lies lateral to the sphincter complex.

**Figure 1:**
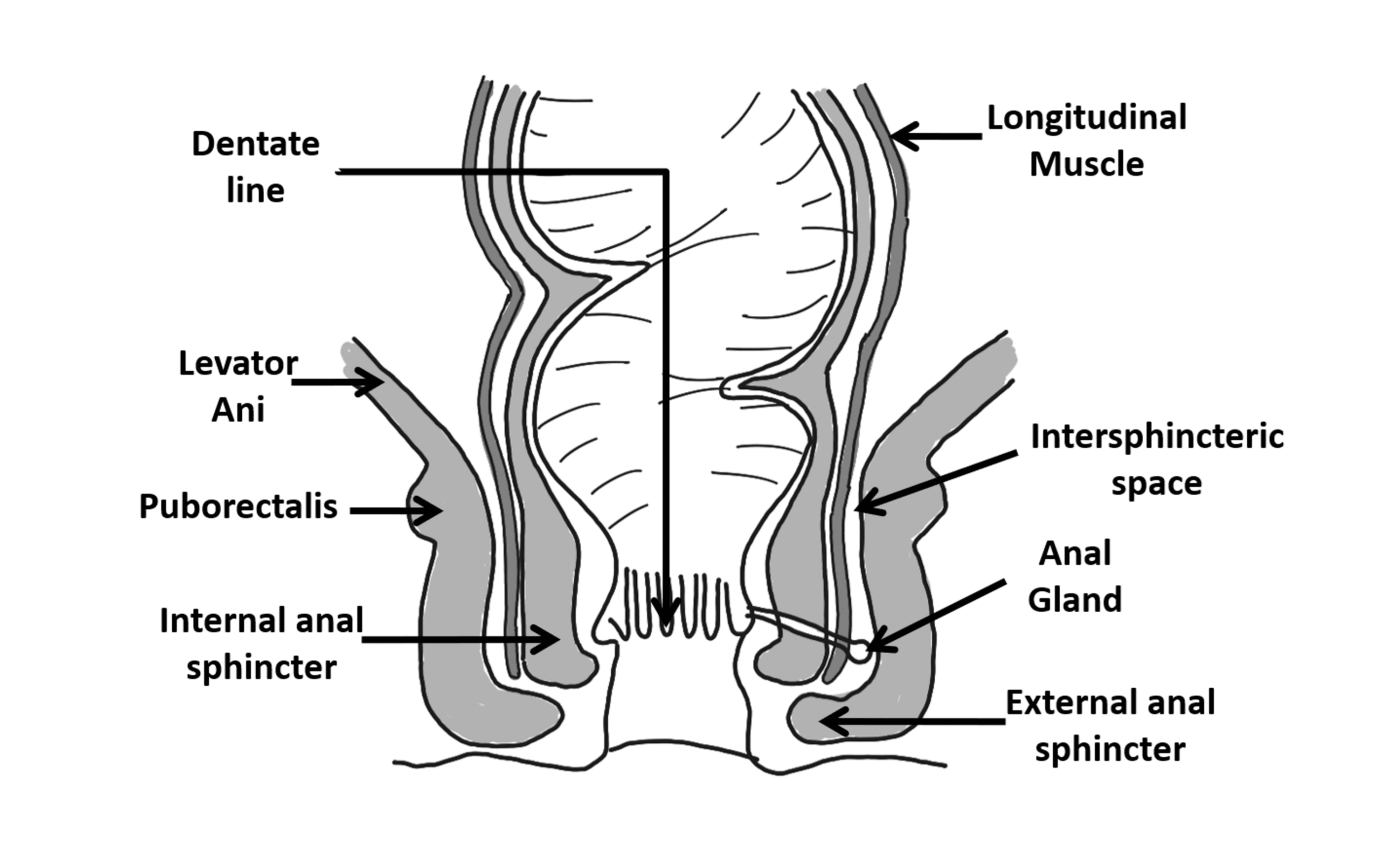
Anatomy of the anal canal in the coronal plane We hereby state that Figure 1 is an original image created by the authors.

The proximal half of the anal canal contains longitudinal mucosal folds, the anal columns of Morgagni. The distal aspect of each column is linked to its neighbor by a small semilunar fold (the anal valves), which in turn forms small pockets (the anal sinuses, or crypts of Morgagni). At the distal undulating end of these valves is the dentate (pectinate) line, which also marks the most distal aspect of the anal transitional zone, a histologic junction between the anal squamous epithelium and rectal columnar epithelium. The dentate line lies approximately 2 cm proximal to the anal verge and is a crucial landmark since the anal glands empty into the crypts that lie proximal to the valves. The origin of the anal glands within the surrounding tissues is variable. They may be present in the subepithelium or the internal sphincter, and approximately one- to two-thirds of these glands are deeply seated within the intersphincteric space (Figure 1) [4].

It is believed that infection of these results in the formation of anorectal sinuses and fistulas, in a process known as the “cryptoglandular hypothesis” [5]. Also, lymphoid aggregates surround the anal glands, which may partly explain the increased incidence of anal fistula in Crohn's disease [6,7].

**Figure 2:**
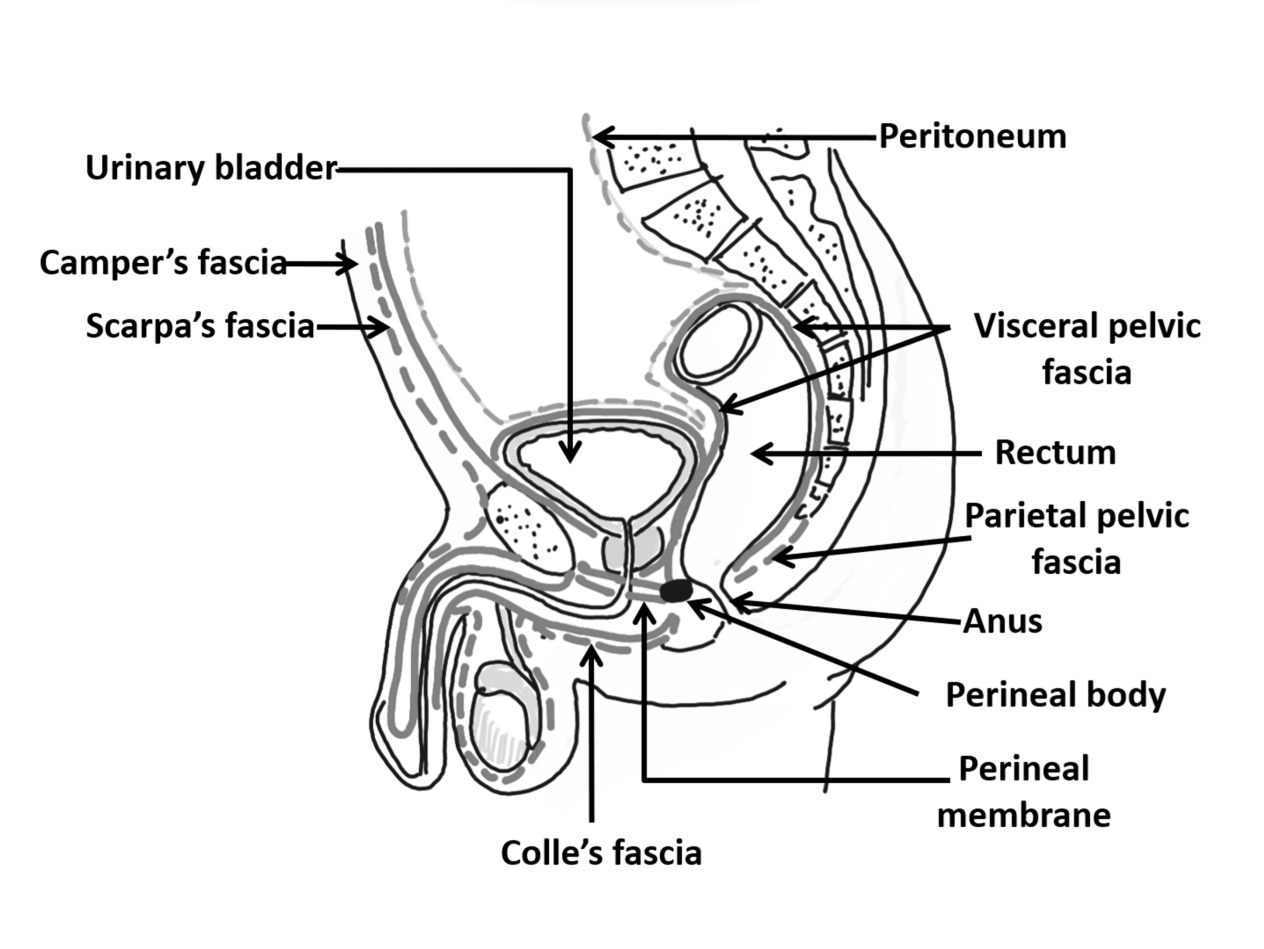
Anatomy of the male pelvis along with major pelvic and perineal fasciae (sagittal view) We hereby state that Figure 2 is an original image created by the authors.

The anatomy of the male pelvis along with major pelvic and perineal fasciae is depicted in Figure 2. It is to be noted that this figure will be used for schematic illustration of the radiological findings in our case series.

Case 1

A 22-year-old man, who was a follow-up case of severe steroid refractory ulcerative colitis (UC), was referred to our hospital. He had undergone total proctocolectomy and end-ileostomy four years back, followed by exploratory laparotomy and adhesiolysis six months after initial surgery for subacute intestinal obstruction and urinary retention. Stoma closure for the patient was attempted eight months after adhesiolysis but failed due to the short length of the stoma. At the time of presenting to our hospital (three years after failed surgical attempt), he had mucus and mild watery discharge from the anal opening for one month. Digital rectal examination (DRE) revealed a hard mass along the anterior anal wall. CT enterography revealed an ill-defined heterogeneously enhancing retroperitoneal soft tissue lesion in the presacral space, for which MRI was done.

**Figure 3:**
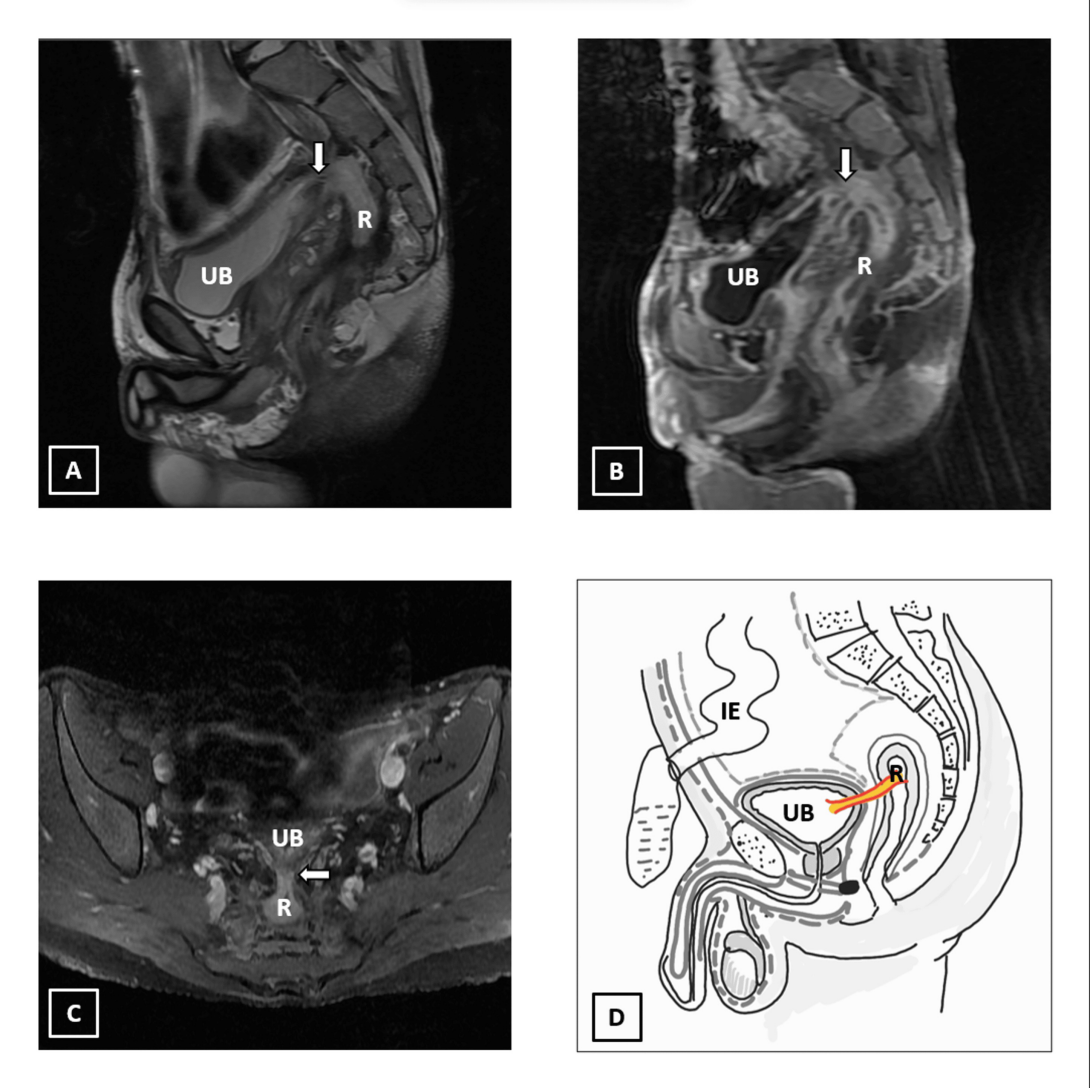
Fistulous communication (arrow) between the posterior urinary bladder wall and upper rectal stump in a 22-year-old male, who is a known case of steroid-refractory severe ulcerative colitis and had previously undergone proctocolectomy and end-ileostomy four years ago. A) Sagittal T2 FSE. B) Sagittal T1 fat saturated post-contrast LAVA. C) Axial T1 fat saturated post-contrast FSE. D) Schematic diagram of the fistulous communication. UB: urinary bladder; R: rectal stump; IE: end-ileostomy. We hereby state that Figure 3D is an original image created by the authors.

MRI revealed a fistulous tract between the posterior bladder wall and upper rectal stump. The fistula shows inflammatory bulbous thickening of size ~ 14 x 16 mm in the midline pre sacral area. Associated inflammatory changes in the form of fat stranding are noted around the tract (Figure 3).

The fistulous connection was successfully repaired using a trans-perineal approach under spinal anesthesia. He was informed that closing the ileostomy with an ileo-rectal anastomosis might not be feasible due to fibrosis and shortening of the mesentery and bowel loops. This condition has made it difficult to mobilize the ileal loop, which means he may need to live with the end-ileostomy for the rest of his life. After the repair of the fistulous communication, the patient is kept on follow-up and is doing well.

Case 2

A 29-year-old-male patient was having multiple episodes of intermittent pain in the perianal region and aphthous oral ulcers for one year. Lower GI endoscopy which was done a year ago revealed inflammatory changes in the ileo-caecal junction and rectum with fistulous anal tract at 6 o'clock position in the anal canal. Biopsy from the inflamed bowel wall revealed active colitis with microgranulomas on histopathology. The patient was diagnosed as a case of Crohn’s disease and managed conservatively on non-steroidal immune-modulators (mesalamine, azathioprine, and infliximab). Currently, the patient presented with complaints of urgency, dribbling of urine, and hesitancy. There was passage of urine per-rectum while micturating. There was also mild pain with intermittent pus discharge per rectum.

**Figure 4:**
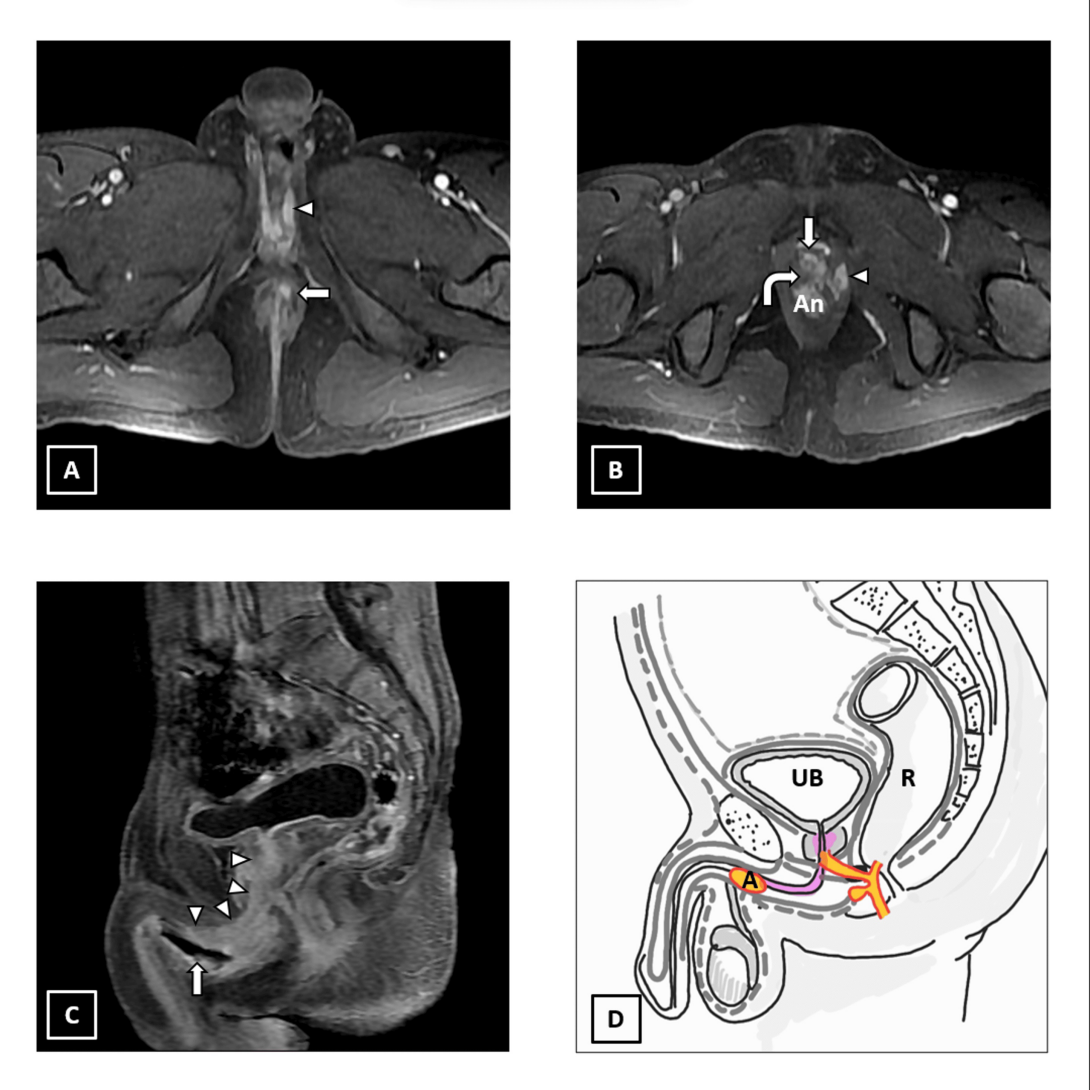
A 29-year-old male patient, with a known case of Crohn’s disease with complex perianal fistula. A) Axial T1 fat saturated post-contrast LAVA sequence showing the caudal aspect of the perianal fistula having inter-sphincteric course (arrow). Note: inflammatory changes in the corpus spongiosum (arrowhead). B) Axial T1 fat saturated post-contrast LAVA sequence showing the cranial aspect of inter-sphincteric fistula where it shows internal opening into the anal canal (An) at 12 o’clock position. A ramification (curved arrow) seen at this level is extending anteriorly into an inflamed prostatic urethra with fistulous opening (arrow). Another ramification at this level is extending left laterally into the left pubo-rectalis muscle (arrowhead). C) Sagittal T1 fat saturated post-contrast LAVA sequence showing inflammation along prostatic, membranous and proximal penile urethra (arrowheads). An air-filled abscess is seen within corpus spongiosum (arrow). D) Schematic diagram of the complex perianal fistula. UB: urinary bladder; A: corpus spongiosum abscess; R: rectum; ‘area shaded in pink’: urethral and periurethral inflammation. We hereby state that Figure 4D is an original image created by the authors.

MRI revealed a complex perianal inter-sphincteric fistulous tract with an external cutaneous opening and two ramifications. One of the ramifications was extending into the left puborectalis muscle, while the other was extending into the prostatic urethra forming fistulous communication. There is an extension of inflammation along the membranous urethra up to the penile urethra with abscess formation in the corpus spongiosum (Figure 4).

Supra-pubic catheterization was performed to divert urine and allow the ano-urethral fistula to heal. The patient received antibiotic therapy, which included nitrofurantoin, ciprofloxacin, and tinidazole. The patient's symptoms gradually improved. In follow-up visits, the fistula healed, and the supra-pubic catheter was removed. The patient is now on non-steroidal immunomodulators for the primary disease. During the follow-up, the patient is doing well.

Case 3

A 36-year-old man presented with left sided scrotal pain (gradual onset and progressive over six days). There was associated tenderness in the root of scrotum and perineal region. Local USG revealed left sided funiculitis and epididymitis with perineal inflammatory changes. MRI was done to look for extent of perineal inflammation.

**Figure 5:**
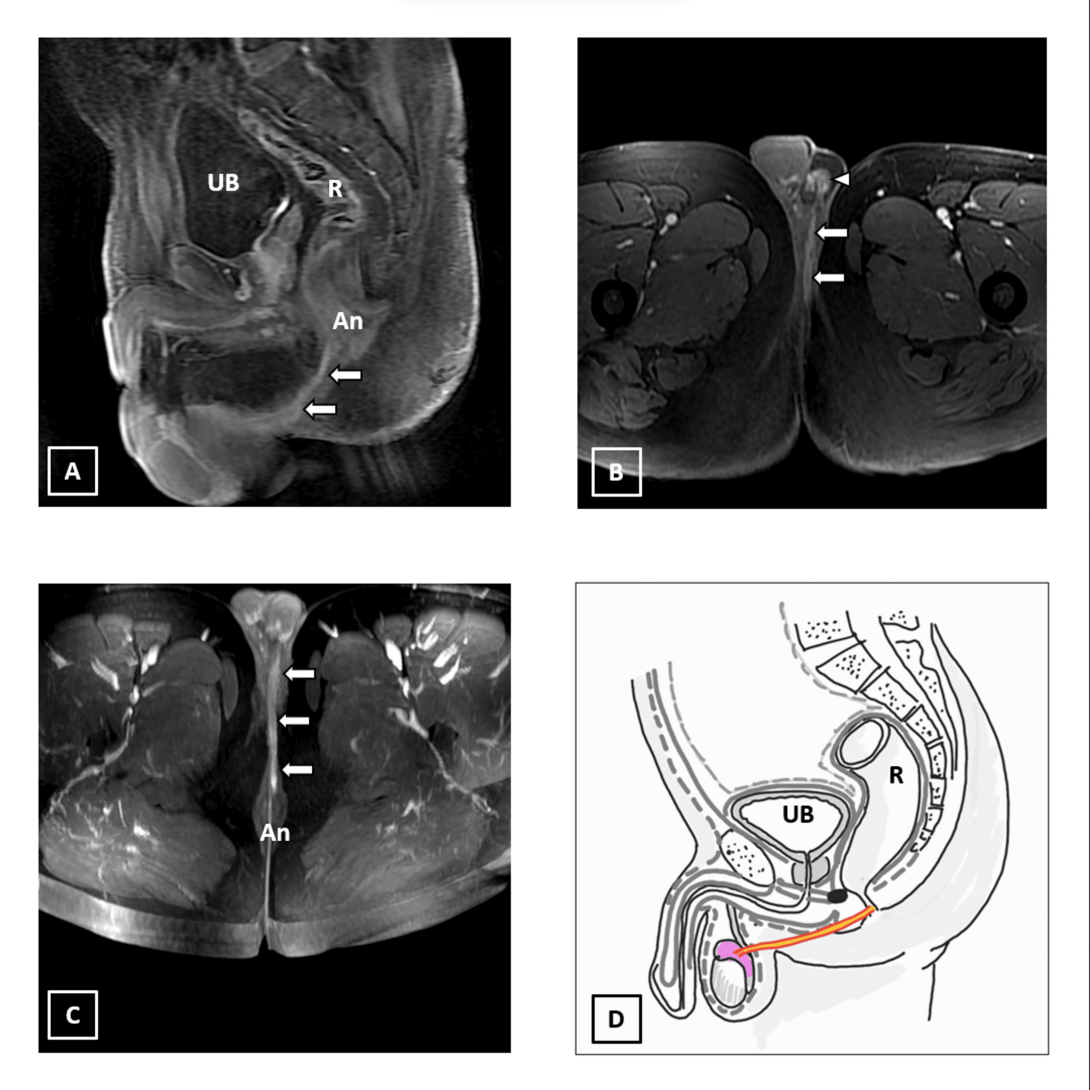
A 36-year-old man with left acute scrotal pain and perineal tenderness. A) Sagittal T1 fat saturated post-contrast LAVA sequence showing blind ending tract (arrows) from the lower anal canal (An) to left scrotal sac. B) Axial T1 fat saturated post-contrast LAVA sequence showing an enhancing perineal tract (arrows) and enhancing inflamed left epididymis (arrowhead). C) Oblique axial MIP of T1 fat saturated post-contrast LAVA sequence showing the entire length of enhancing blind ending tract (arrows) from the lower anal canal (An) to left scrotal sac. D) Schematic diagram of the perianal tract with left epididymitis (shaded in pink). UB: urinary bladder; R: rectum We hereby state that Figure 5D is an original image created by the authors.

MRI revealed a blind ending tract arising from anterior wall of lower anal canal near the anal verge and extending into the left scrotal sac with associated thickening and inflammation of the epididymis (Figure 5).

Fistulectomy was performed under general anesthesia, after which the patient was started on antibiotics for two weeks. The patient showed clinical improvement with resolution of scrotal tenderness.

Case 4

A 20-year-old man, with a known case of Crohn’s disease and Takayasu arteritis and a past history of tuberculosis, presented with complaints of loose stools (six episodes/ day) which were watery in consistency and not associated with pus or bloody discharge. There was mild swelling and redness in the bilateral scrotum.

**Figure 6:**
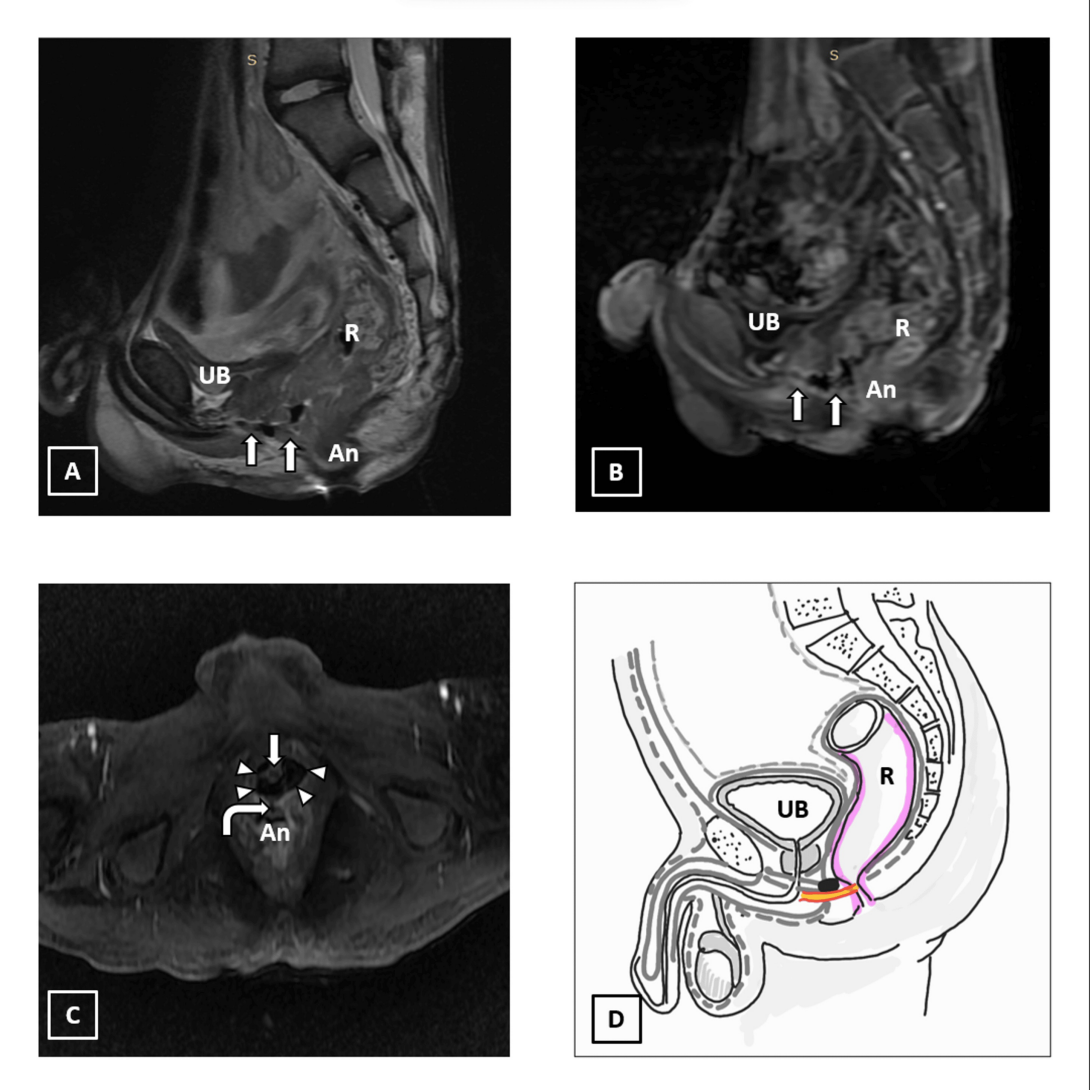
A 20-year-old man, with a known case of Crohn’s disease and Takayasu arteritis and a past history of tuberculosis, presenting with watery diarrhea and bilateral mild scrotal swelling and redness. A) Sagittal T2 image showing air-filled tract extending from the anal canal (An) to the root of the scrotum (arrows). There is circumferential thickening of rectal (R) and anal canal walls. B) Sagittal T1 fat saturated post-contrast LAVA sequence showing an inflamed perianal tract as mentioned above (arrows) with inflammatory hyperenhancement along the tract walls, as well as thickened rectal (R) and anal (An) walls. C) Axial T1 fat saturated post-contrast LAVA sequence showing the perianal tract extending from the anus (An) to the root of the scrotum with encasement of membranous urethra (arrow) in a horse-shoe fashion (arrowheads). D) Schematic diagram of perianal tract reaching up to the root of the scrotum. Area shaded in pink: anorectal inflammation; UB: urinary bladder We hereby state that Figure 6D is an original image created by the authors.

MRI revealed an air-filled tract extending from the anal canal anteriorly up to the root of the penis. Diffuse thickening of the sigmoid colon, rectum, and anal canal wall was seen with associated inflammatory changes and fascial thickening. The tract appears to be encasing the membranous urethra near the root of the penis in a horse-shoe shaped manner. There is mild inflammation of membranous urethra (Figure 6).

After reviewing the case our gastro-surgery team opined that there was spontaneous fibrosis and healing of the fistulous tract ruling out the need for active surgical intervention. The patient was conservatively managed on antibiotics (ciprofloxacin and metronidazole) following which his genitourinary symptoms improved. Anti-tubercular treatment and mesalazine were continued for the patient.

## Discussion

The four cases presented in our paper are compared in Table 1.

**Table 1:** Comparative study of our four cases

Case No.	Age	Presenting complaints	Contributing illness with duration	Imaging findings
Case 1	22 years	Mucus/ mild watery discharge from the rectal stump (Status: Post proctocolectomy and end-ileostomy)	Steroid-resistant ulcerative colitis x six years	Fistulous communication between the urinary bladder and rectal stump.
Case 2	29 years	Urgency, dribbling of urine, and hesitancy Passage of urine per-rectum while micturating	Crohn’s disease for the last one year	Complex perianal fistula with external opening as well as fistulous opening into urethra, with urethritis and corpus spongiosum abscess
Case 3	36 years	Left-sided scrotal pain	None identified	Blind ending tract extending from anus to left scrotal sac with left epididymitis
Case 4	20 years	Loose stools with mild swelling and redness in bilateral scrotum	Known case of Crohn’s disease and Takayasu arteritis and past history of tuberculosis	Perianal tract extending from anus to root of the penis with horse-shoe-shaped encasement of the membranous urethra and mild urethritis

UC predominantly causes inflammation of the mucosa. This pathophysiology indicates that UC does not normally form fistulas with other organs [8]. However, fistulas may form due to other diseases complicated with UC such as diverticulitis or cancer. In Case 1, no such other predisposing diseases besides UC were found. Rectovaginal fistulas complicating UC, though rare, have been reported [9]. However, there was hardly any literature reporting rectovesical fistula in UC as in Case 1. The possibility that the fistula may have been formed due to operative complications, is unlikely because the patient presented with watery discharge three years after the last surgery.

Fistulous communication between the bowel and urethra, though rare, has been reported in the literature [10]. In Case 2, the ano-urethral fistula was clinically diagnosed as the patient had symptoms similar to a lower urinary tract infection with the passage of urine per-rectum while micturating.

In Case 3, the patient was clinically diagnosed as a case of acute scrotum. Scrotal USG revealed left epididymitis and funiculitis. The additional finding of perineal inflammatory change prompted a pelvic MRI to look for the extent of the inflammation, which led to the discovery of the perianal tract. Cases of perianal/ perirectal tracts, presenting with epididymitis, though rare, have been previously reported [11].

Perianal fistulous or sinus tracts with scrotal extensions have been described in the literature [12]. Case 4 shows similar findings with a horse-shoe-shaped abscess partially encasing the membranous urethra. Horse-shoe-shaped abscesses surrounding the anus have been commonly reported [13,14]. However, the authors are unaware of any case report of such abscess surrounding the urethra or other pelvic hollow viscera, as has been described in Case 4.

The review of previous literature shows a scarcity of reported cases of acquired ano-rectal sinuses and fistulas involving genitor-urinary structures in men. All four cases reported by us were young adults less than 40 years of age. Three out of the four cases had a history of inflammatory bowel disease. All the cases present with atypical symptoms with clinical signs of genito-urinary disease.

## Conclusions

Involvement of genito-urinary structures by acquired ano-rectal sinuses and fistulas in men is a rare and underreported phenomenon. These cases often present with nonspecific or unusual symptoms, making early recognition difficult. Inflammatory bowel disease patients with persistent genito-urinary complaints and poor response to antibiotics should be evaluated for the possibility of a complex ano-rectal fistula or sinus. Early evaluation with pelvic MRI can be crucial in identifying these conditions and facilitating appropriate treatment.
